# Graphene-Type Materials for the Dispersive Solid-Phase Extraction Step in the QuEChERS Method for the Extraction of Brominated Flame Retardants from *Capsicum* Cultivars

**DOI:** 10.1021/acs.jafc.2c07873

**Published:** 2023-02-16

**Authors:** Virgínia Cruz Fernandes, Valentina F. Domingues, Marta S. Nunes, Renata Matos, Iwona Kuźniarska-Biernacka, Diana M. Fernandes, Antonio Guerrero-Ruiz, Inmaculada Rodríguez Ramos, Cristina Freire, Cristina Delerue-Matos

**Affiliations:** † REQUIMTE/LAQV, Instituto Superior de Engenharia do Porto, Instituto Politécnico do Porto, Rua Dr° António Bernardino de Almeida, 431, 4249-015 Porto, Portugal; ‡ REQUIMTE/LAQV, Departamento de Química e Bioquímica, Faculdade de Ciências, Universidade do Porto, Rua do Campo Alegre s/n, 4169-007 Porto, Portugal; § Dpto. Química Inorgánica y Técnica, Facultad de Ciencias UNED, Senda del Rey 9, 28040 Madrid, Spain; ∥ Instituto de Catálisis y Petroleoquímica, CSIC, Cantoblanco, Marie Curie 2, 28049 Madrid, Spain

**Keywords:** graphene-type material sorbents, peppers, Capsicum cultivars, QuEChERS, flame retardants

## Abstract

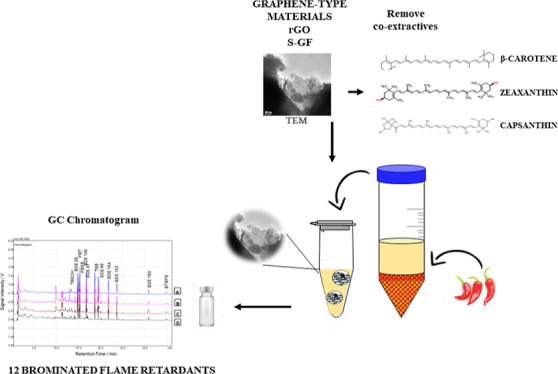

A new application of graphene-type materials as an alternative cleanup sorbent in a quick, easy, cheap, effective, rugged, and safe (QuEChERS) procedure combined with GC–ECD/GC–MS/GC–MS/MS detection was successfully used for the simultaneous analysis of 12 brominated flame retardants in *Capsicum* cultivar samples. The chemical, structural, and morphological properties of the graphene-type materials were evaluated. The materials exhibited good adsorption capability of matrix interferents without compromising the extraction efficiency of target analytes when compared with other cleanups using commercial sorbents. Under optimal conditions, excellent recoveries were obtained, ranging from 90 to 108% with relative standard deviations of <14%. The developed method showed good linearity with a correlation coefficient above 0.9927, and the limits of quantification were in the range of 0.35–0.82 μg/kg. The developed QuEChERS procedure using reduced graphite oxide (rGO) combined with GC/MS was successfully applied in 20 samples, and the pentabromotoluene residues were quantified in two samples.

## 1 Introduction

Brominated flame retardants (BFRs) are usually included as a chemical additive used in a variety of consumer goods and everyday objects like insulation materials, plastic electronic devices, textiles, food containers, etc. Although the use of certain BFRs was banned and restricted by the European Union due to their persistence and bioaccumulation in the environment, they were detected in biota, food, and feed samples.^1−5^

Although BFR levels found in food samples are mostly associated with environmental contamination, the potential influence from packaging cannot be excluded. A wide range of plastic products may contain BFRs. Material recycling may potentially reintroduce the BFR into new plastic product cycles and lead to increased exposure levels, for example, through the use of plastic packaging materials.^6,7^

Consumption of BFR-contaminated food products may result in serious health problems in human beings.^8−14^ The toxicity of these plastic additives to humans demonstrated that BFRs have the potential to adversely affect endocrine functions and the central nervous and reproductive systems. The different brominated diphenyl ethers (BDE) appear to have a similar toxicological profile, with the liver, kidney, and thyroid as the main target organs.^15^ Several analytical techniques are used in monitoring studies of pesticides and other contaminants in food samples.^16−18^ These include mainly liquid chromatography and gas chromatography for multiple contaminant analysis and electrochemical techniques usually for one target.^19,20^

Some BFRs are present in complex matrices in vestigial amounts, such that they cannot be simply detected. Complex samples are challenging and require more attention, and many limitations need to be explored to mitigate matrix effects that analytical chemists must overcome. Thus, challenges can be met by skillful sample pre-concentration of several compounds simultaneously prior to target analyses. The approaches should be tailored to study matrix interferences and produce effective, reliable, and accurate methods for extraction and analysis. The *Capsicum* cultivars are samples that have a complex composition since they contain numerous chemicals including steam-volatile oils, fatty oils, capsaicinoids, carotenoids, vitamins, proteins, fibers, and mineral elements.^21^ Sample preparation techniques for BFR extraction include QuEChERS,^1^ dispersive liquid–liquid micro-extraction, solid-phase extraction (SPE), solid-phase micro-extraction, and other approaches use adsorbent-based techniques for testing new synthesized materials such as QuEChERS with magnetic nanoparticles.^22^ These new trends of procedures, using magnetic nanoparticles, have evolved, gained visibility, and grown due to the successful results. In addition, the main attributes were organic solvent-free/reduction, small amounts of adsorbents, fewer steps, and less time and energy.^22^ The authors also showed that these magnetic nanoparticle adsorbents, mainly the ones with particles with a 200 nm average size, presented an adsorption ability toward pigment matrix and other co-extracts from strawberry samples. This type of adsorbent has also shown a set of successful applications when applied in the pre-concentration of BFRs in other red fruit samples (blueberry and raspberry).^2^ Some authors reported the use of graphene materials in several extraction methods for pesticide analysis in food samples.^23−25^ Recently, Zheng et al. reported the synthesized and successful use of ZIF-8@nitrogen-doped reduced graphene oxide as a coating material for the extraction of halogenated flame retardants in crayfish.^26^ Another application using graphene oxide-based surface molecularly imprinted polymers was reported for organophosphate flame retardants in environmental water.^27^ Khetagoudar et al. reported the excellent performance of a graphene nanocomposite in the elimination of pigments in green chili matrices.^28^ To the best of our knowledge, studies in which graphene-type materials [reduced graphite oxide (rGO) and sulfur-doped graphene (S-GF)] are applied for QuEChERS extraction of BFRs from fruits/vegetables are not reported in the literature. It is, therefore, imperative to innovate and develop new methods to meet the needs for sample integrity, rapid analysis, cost-effective, and environmentally friendly methods, precision, and reproducibility. Also, no MRLs have been established for BFR, but the European Commission recommended their monitoring in food.^29^

Here, for the first time, an analytical approach developed to meet the demands using different dispersive SPE (d-SPE) sorbents, namely, synthesized graphene-type materials (rGO and S-GF), was evaluated to meet the challenge of removing the interferents from a complex food sample (*Capsicum* cultivars) with a high content of pigments (carotenoids), spicy compounds (capsaicinoids), fatty oils, protein, mineral elements, and so forth. Moreover, the possible contamination of the target analytes [polyBDE (PBDE) congeners and novel BFR] conditioned in plastic packaging was also explored.

## 2 Materials and Methods

### 2.1 Reagents

The standards of BFRs (seven PBDE congeners and five novel BFRs) with high purity (≥97%) were purchased from Isostandards Material, S.L. (Madrid, Spain) and stored in a freezer. The 12 compounds were 1,2-dibromo-4-(1,2-dibromoethyl)-cyclohexane (TBECH), pentabromotoluene (PBT), 2,4,4′-tribromodiphenyl ether (BDE28), pentabromoethylbenzene (PBEB), 2,2′,4,4′-tetrabromodiphenyl ether (BDE47), 2-ethylhexyl 2,3,4,5-tetrabromobenzoate (TBB), 2,2′,4,4′,5-pentabromodiphenyl ether (BDE99), 2,2′,4,4′,6-pentabromodiphenyl ether (BDE100), 2,2′,4,4′,5,5′-hexabromodiphenyl ether (BDE153), 2,2′,4,4′,5,6′-hexabromodiphenyl ether (BDE154), 2,2′,3,4,4′,5′,6-heptabromodiphenyl ether (BDE183), and 1,2-bis(2,4,6-tribromophenoxy)ethane (BTBPE). The internal standard (IS) 5′-fluoro-2,3′,4,4′,5-pentabromodiphenyl ether was also obtained from Isostandards Material, S.L. (Madrid, Spain). The *n*-hexane UniSolv was supplied by Merck (Darmstadt, Germany), and the acetonitrile was obtained from Carlo Erba (Val de Reuil, France). The QuEChERS EN method and bulk sorbents, namely, primary secondary amine (PSA), C18-silica, and carbon, were obtained from Agilent Technologies (California). The carbon bulk specifications described that the average particle size was 40–120 μm and irregular. A working standard mixture of the contaminants containing a total of 12 BFRs was prepared at 150 μg/L in *n*-hexane. This mixture was used to prepare spiked and calibrated standard solutions in *n*-hexane. Data and statistical analysis were carried out using GraphPad Prism 6.0 and Excel software.

### 2.2 Preparation and Characterization of Graphene-Type Materials

The graphene-type materials were prepared by procedures already reported.^30,31^ Briefly, the rGO preparation consisted, in the first step, the addition of 10 g of graphite and over 200 mL of fuming HNO_3_, keeping the mixture at 0 °C. Then, 80 g of KClO_3_ was gradually added over 2 h; afterward, the mixture was stirred for 21 h, maintaining the temperature of 0 °C. The graphene (GO) material obtained was filtered and washed with water until a neutral pH was reached and then dried under vacuum at room temperature. In the second step, the GO was exfoliated in a vertical quartz reactor under a nitrogen atmosphere by heating at 10 °C/min to 250 °C and keeping this temperature for 30 min; then, the temperature was increased from 250 to 700 °C using the same heating rate, and then this temperature was maintained for 30 min. At the end of the process, the rGO material was obtained.^31^ The S-GF was prepared by milling 0.5 g of commercial graphene flakes (GF) together with 0.15 g of elemental sulfur in ball milling equipment (Retsch, MM200) for 5 h at a frequency of 15 s^–1^ using two zirconium oxide balls (1.5 cm in diameter). The obtained material was then pyrolyzed in a nitrogen atmosphere, heated up to 600 °C at a heating rate of 2 °C/min (Nabertherm), and maintained at this temperature for 1 h.^30^

The prepared materials were characterized by several techniques, such as Raman, XPS, XRD, SBET, and TEM. All details (Materials and Methods) regarding these can be found in the Supporting Information file.

### 2.3 *Capsicum* Cultivar Samples

A total of 20 samples of *Capsicum (C.) annuum* L., *Capsicum frutescens* L., and *Capsicum chinense* (packaged and stored in plastic containers) were purchased in Porto, Portugal, at local supermarkets. The samples were separately frozen at −20 °C. Each sample was coldly homogenized using a high-performance blender (Vorwerk, Portugal). The grinding of the sample was performed at the maximum speed of the equipment (speed 10) for 10 s; then, the sample was removed from the walls of the container, and the sample was ground again at a speed of 10 for 10 s. After this sample preparation, the samples were frozen and stored in the same package until extraction and analysis.

### 2.4 Sample Preparation Method

Samples (10 g) were mixed with 10 mL of acetonitrile in a 50 mL Falcon tube. Subsequently, EN QuEChERS salts were added, and the sample was vortexed for 1 min. The tube was centrifuged for 5 min at 4500 rpm. 1.5 mL of the upper layer was transferred to a 2 mL microtube with the addition of different sets of sorbents (Table 1). The microtube was shaken and vortexed for 1 min. After centrifugation at 4500 rpm for 5 min, 1 mL of the supernatant was transferred into a 1.5 mL vial. The sample was dried with a gentle flow of nitrogen gas and redissolved in the same volume of *n*-hexane. The IS was added at a constant concentration for analytical quality control immediately before injection. After vortexing, the sample was ready for gas chromatographic analysis.

**Table 1:** Cleanup Set Composition

cleanup sets	MgSO_4_	PSA	C18	carbon	rGO	S-GF
CL1	150	50	50	50		
CL2	150	50	50	2		
CL3	150	50	50	5		
CL4	150	50	50		2	
CL5	150	50	50		5	
CL6	150	50	50			2
CL7	150	50	50			5

#### 2.4.1 Method Validation

The analytical performance of the gas chromatography system with an electron capture detector (GC-ECD) was carried out through the following parameters based on SANTE/11813/2017^32^ and EURACHEM guidelines:^33^ linearity with a matrix-matched calibration curve, matrix effect, recoveries, the limit of detection (LOD), the limit of quantification (LOQ), and repeatability and reproducibility. The LOD and LOQ were calculated based on the standard deviation of the signal and the slope of the calibration curve. The matrix effects and intra-day and inter-day precision were calculated according to Fernandes et al. 2020.^2^ The *C. annuum* L. extract spiked with each of the BFRs at seven concentration levels (μg/kg) (ranging between 0.10 and 1.88 μg/kg) was prepared for the establishment of the calibration curve. For each level, the determinations were performed in triplicate under optimal conditions. The recovery and repeatability of 12 BFRs were determined to evaluate the method’s performance in *C. annuum* L. The repeatability and trueness of the method were studied by carrying out six consecutive extractions at three concentration levels (0.38, 0.75, and 1.13 μg/kg).

### 2.5 GC-ECD, GC/MS, and GC/MS/MS Analyses

Analysis was performed on GC-ECD (GC-2010, Shimadzu, Quioto, Japan) equipped with a Zebron-5MS fused silica capillary column (30 m × 0.25 mm i.d. × 0.25 μm film thickness) (Phenomenex, Madrid, Spain) used for separation and ultrapure helium (purity ≥ 99.999%) used as the carrier gas at a flow rate of 1.0 mL/min. Chromatographic data were recorded and processed with Shimadzu’s GC Solution software. The parameters were according to those.^1^ Briefly, the gas chromatography conditions were as follows: the injector temperature was 250 °C; the initial oven temperature was 90 °C (held for 1 min), which was then increased to 150 °C at 16 °C/min (held for 1 min) and then to 290 °C at 16 °C/min (held for 30 min); and the gas chromatography detector was maintained at 300 °C. The splitless injection was adopted throughout the whole experiment. The method validation and all the analyses were performed in GC-ECD. GC/MS analysis with a Trace-Ultra GC (Thermo Fisher Scientific, Waltham, USA) coupled to an ion trap mass detector, Thermo Polaris, was performed at the same conditions as GC-ECD in all the positive samples observed in GC-ECD in order to have confirmation. Data acquisition was performed first in the full scanning mode from 50 to 500 *m*/*z* to confirm the retention times of the analytes. All standards and sample extracts were analyzed in the selective ion monitoring (SIM) mode and tandem mass spectrometry (MS/MS). The SIM ions selected for the PBT confirmation were 486, 328, and 326, and the precursor ions for MS/MS were 486 and 326.

### 2.6 Statistical Analysis

ANOVA statistical analysis was performed to estimate significant differences among different analytical procedures using GraphPad software.

## 3 Results and Discussion

### 3.1 Characterization of Graphene-Type Materials

Even though these two graphene-type materials were previously prepared and their characterization was reported in the literature,^30,31^ here, a summary of the main aspects of their characterization is given for the sake of the reader to avoid labyrinthic access to data. The rGO was characterized by several techniques, namely, N_2_-adsorption isotherms, XRD, TEM, XPS, and Raman spectroscopy.^31^ The surface area determined by the BET method to N_2_ adsorption isotherms (Figure S1a, type IV isotherms) was 867 m^2^ g^–1^, which suggests the formation of a few-layer graphene structure for rGO. The average pore diameter was found to be 7.9 nm by the BJH analysis (Figure S2a). The XRD corroborated the formation of a few-layer graphene structure (average stacking number of 12 layers) with an interlayer distance of 0.34 nm. The rGO TEM micrograph (Figure S3a) showed the presence of winkled structures of graphene consisting of 5–12 graphene layers. The characterization of the rGO composition by XPS analysis (Figure S4a) indicated a surface composed of carbon and oxygen (93 and 7%, respectively). Raman spectroscopy is widely used for the characterization of carbon materials (graphitic materials, carbon fibers, glassy carbon, fullerenes, carbon nanotubes, graphene, etc.), especially because conjugated and double carbon–carbon bonds lead to high Raman intensities.^34^ The stretching of the C–C bond in graphitic materials gives rise to the so-called G-band Raman feature, which is common to all sp^2^ carbon systems (∼1580 cm^–1^). The G-band is highly sensitive to strain effects in sp^2^ nanocarbons and can be used to probe any modification to the flat geometric structure of graphene. The D band (∼1350 cm^–1^) is significant in providing information about the electronic and geometrical structures through the double resonance process.^34^ The intensity ratio between the D and G bands, *I*_D_/*I*_G_, is often used to estimate the disorder degree of graphitic materials.^35,36^

By Raman spectroscopy, the degree of disorder in the structure of graphene was studied using the intensity ratio of the D and G bands (*I*_D_/*I*_G_) as a quantitative indicator of the amount of disorder or edges of the rGO structure, which was calculated as 0.63.

Regarding the S-GF material, the N_2_-adsorption isotherms (Figure S1b, type IV isotherms) revealed a BET surface area of 284 m^2^ g^–1^, which was appointed to the piling up of some graphene layers in the S-GF structure. The average pore diameter was determined by BJH analysis giving a value of 3.8 nm (Figure S2b). The reported XRD pattern of S-GF indicated an interlayer distance of 0.34 nm and a stacking number of graphene layers of 32, suggesting the formation of a graphenic material with some aggregation of the graphitic layers, following the outputs of the N_2_-adsorption isotherms analyses. The TEM images of S-GF (Figure S3b) showed graphene sheets with some folding mainly at the borders and with sizes in the range of hundreds of nanometers to a few micrometers. The surface composition obtained by XPS (Figure S4b) revealed that S-GF contains 97.5% of C, 1.7% of O, and 0.8% of S, confirming S-doping. The Raman spectroscopy revealed an increase in defect density and disorder due to the S-doping process (in comparison with the original undoped GF), as indicated by the significant increase in the *I*_D_/*I*_G_ ratio obtained for S-GF (1.72 vs 0.61 for undoped GF).

Comparing the rGO and S-GF materials, beyond the S-doping, the S-GF presents a smaller *S*_BET_ than rGO (284 vs 867 m^2^ g^–1^). Although both materials showed mesoporous properties, the rGO has a lower stacking of graphitic layers (*N*_L_ = 12 vs 32 for S-GF), which results in a higher surface area. This difference is most likely due to the completely different starting materials used in their preparation—commercial GF for S-GF and graphite for rGO. The commercial carbon bulk also tested was not characterized. Table S1 summarizes the graphene-type material’s properties.

### 3.2 Optimization of the QuEChERS and d-SPE Procedure

During the QuEChERS procedure, different sets of dispersive sorbents were used in several applications.^2,22^ Cleanup sorbents like PSA, C18, and commercial carbon bulk were commonly applied in the second step of the QuEChERS procedure.^1^ As the matrices are diverse and complex, some work has emerged, namely in the application of new sorbents.^37,38^ Some studies reported that commercial d-SPE, with the use of carbon, presented lower efficiency for planar compounds.^39^

**Figure 1:**
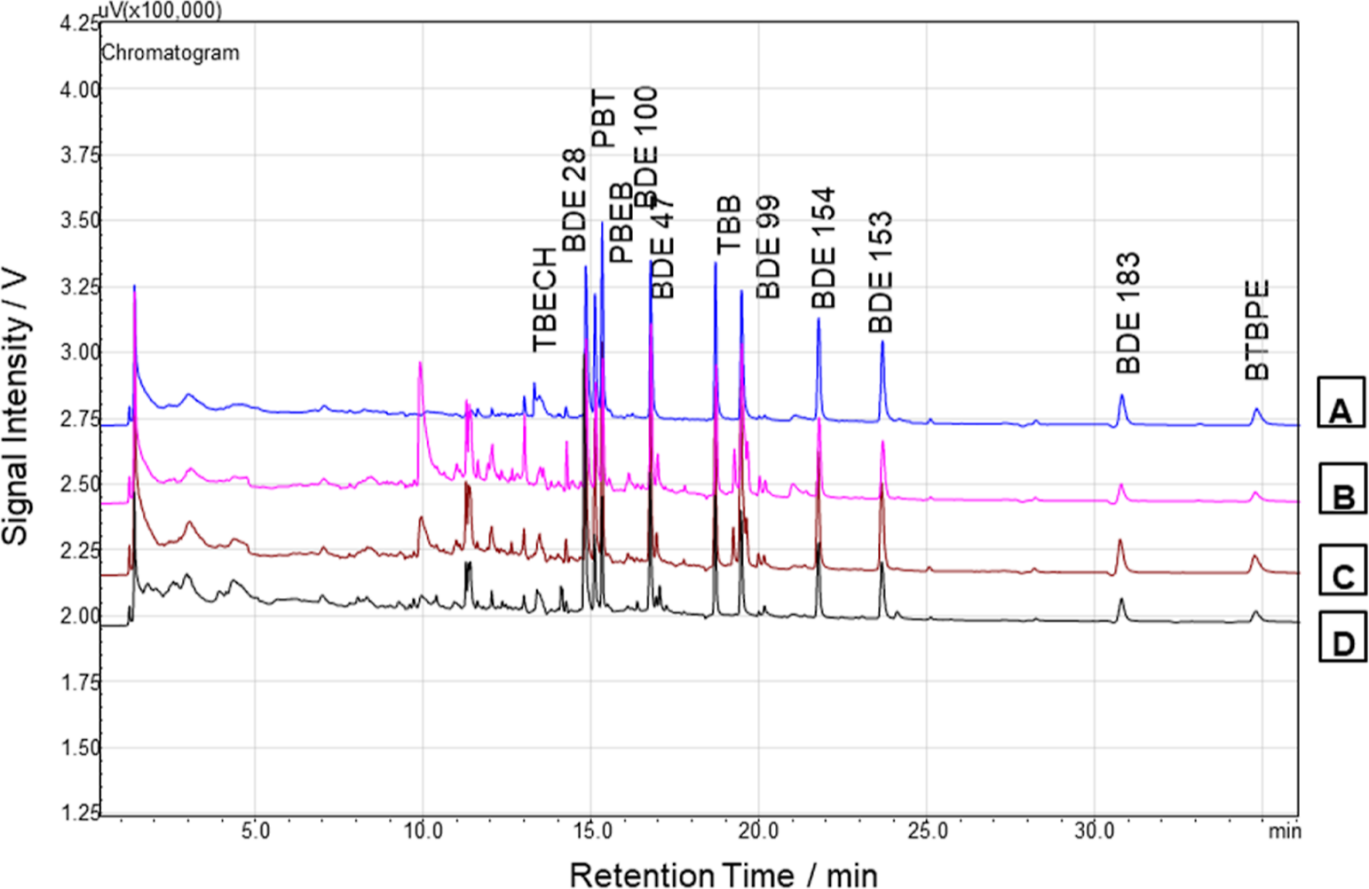
A—BFR mixture standard chromatogram at 50 μg/L. B—chromatograms obtained from an extract of *C. annum* L. spiked at 0.75 μg/kg using the CL3 (150 MgSO_4_ + 50 mg PSA + 50 mg C18 + 5 mg carbon) C—chromatograms obtained from an extract of *C. annum* L. spiked at 0.75 μg/kg using the CL7 (150 MgSO_4_ + 50 mg PSA +50 mg C18 + 5 mg S-GF) D—chromatograms obtained from an extract of *C. annum* L. spiked at 0.75 μg/kg using the CL5 (150 MgSO_4_ + 50 mg PSA + 50 mg C18 + 5 mg rGO).

In the present work, the ability to remove the co-extractive interferences was evaluated using lower amounts of graphene-type material sorbents together with MgSO_4_, C18, and PSA. The chromatograms obtained with different cleanup sets are shown in Figure 1. Chromatogram B revealed that the classic cleanup (MgSO_4_ + PSA + C18 + carbon) is insufficient at removing interferences and during the extraction, the efficiency of the extraction was influenced and reduced. It was observed that chromatogram B compared with C and D shows fewer interferences, and even most of them have disappeared, suggesting that MgSO_4_, PSA, C18, and a small amount of graphene-sorbents effectively remove co-extractive interferences from the matrix. As previously demonstrated by other authors, graphene and other graphene-type materials showed very promising results, even though only studies with pesticides have been reported.^23,26,40−42^

#### 3.2.1 Effect of Dispersive Sorbent Materials

**Figure 2:**
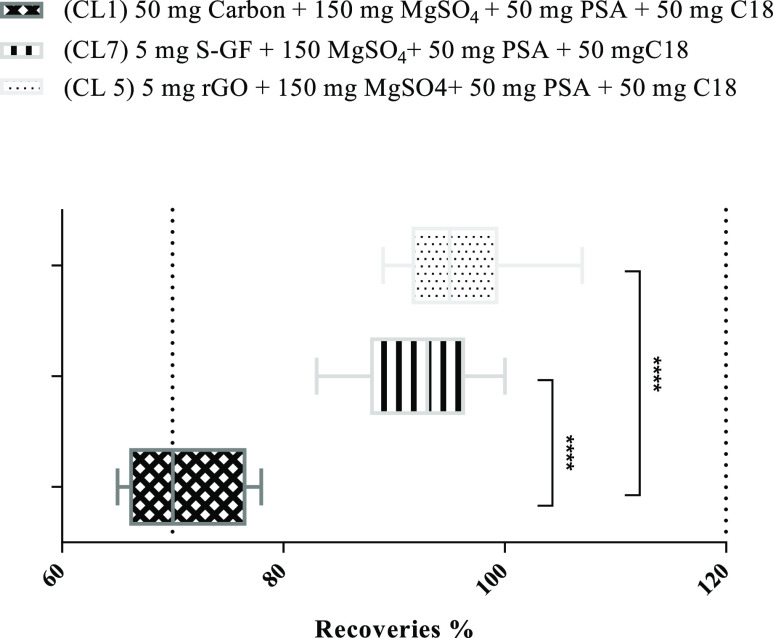
Comparative recovery results between commercial d-SPE with commercial carbon (CL1) and graphene—dispersive sorbent materials (CL5 and CL7). Two-way ANOVA analysis with Sidak’s multiple comparison test (ns-non significant; ***-significant).

The use of different graphene-dispersive sorbent materials (rGO and S-GF) was evaluated during the sample preparation process. To better evaluate the performance of the two types of graphene materials, the extraction efficiency was evaluated through recovery studies. The recovery values obtained for the procedure using graphene materials were compared with those obtained using commercial carbon bulk. Figure 2 shows the average of the recovery values obtained for the experiments comparing graphene-type materials and carbon. The ANOVA statistical analysis was used to compare the average recoveries of each cleanup test. The two-way ANOVA statistical study has shown that the recoveries are significantly different comparing the three different cleanup sets (CL1, CL5, and CL7). Mean values of recoveries increased progressively from cleanup tests using carbon (72%), S-GF (92%), and rGO (96%).

The graphene-dispersive sorbents showed a great improvement in the efficiency of the extraction procedure of the target 12 BFRs. We found that graphene-dispersive sorbents did not adsorb the BFR, while commercial carbon presented some adsorption that was verified by the reduction in recovery. The layered structure of graphene-type materials can explain the advantage of these materials concerning the irregular and disordered structure of commercial carbon. As already reported for graphene,^23,28^ the present graphene-type materials presented not only good removal efficiencies of pigments (extract became colorless after the cleaning step with graphene compounds) in *Capsicum* samples but also excellent cleanup ability for other compounds of the matrix, as you can see in Figure 1. In the literature, a few studies with other graphene-type materials were reported. Luo et al. reported an excellent performance of magnetic graphene (G/PSA/Fe_3_O_4_) as a sorbent in tobacco samples.^42^ The G/PSA/Fe_3_O_4_ presented a transparent, few-layer structure by TEM and SEM with a lower BET surface area.^42^ Other authors reported the excellent performance of a graphene nanocomposite in the elimination of pigments in green chili matrices.^28^ However, these authors have not explored all the morphological characteristics of the nanocomposite, so the studies cannot be compared.

#### 3.2.2 Effect of Graphene—Dispersive Sorbent Amounts

**Figure 3:**
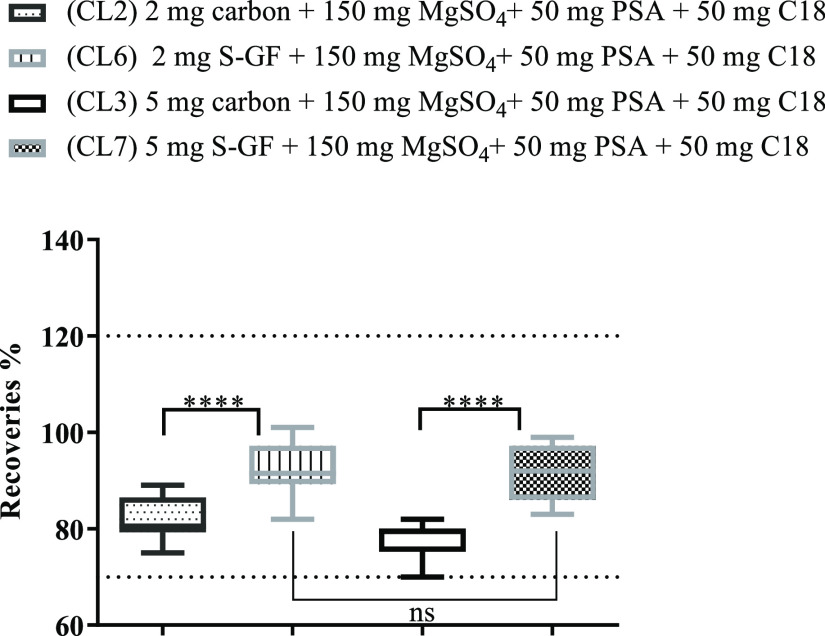
Recovery study comparing four cleanup sets with different amounts of commercial carbon (CL2 and CL3) and S-GF (CL6 and CL7). Two-way ANOVA analysis (ns-non significant; ****-significant)

**Figure 4:**
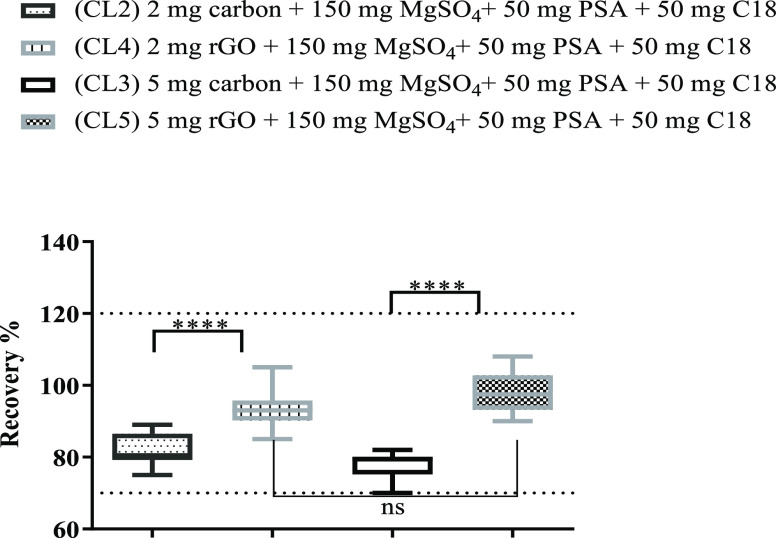
Recovery study comparing four cleanup sets with different amounts of commercial carbon (CL2 and CL3) and rGO (CL4 and CL5). Two-way ANOVA analysis (ns-non significant; ****-significant).

In addition to the differences in the results obtained between different graphene-dispersive sorbents (S-GF and rGO), the amount of each sorbent (2 and 5 mg) was also studied. Figures 3 and 4 show recovery values obtained when two different amounts of sorbents (2 and 5 mg) were tested. The amounts of dispersive sorbents were found to have an influence on the recoveries of the BFR *Capsicum* extracts when compared with d-SPE with commercial carbon. Significant differences were confirmed. However, no significant differences between the recoveries obtained using 2 or 5 mg of graphene sorbents were observed. The chromatograms obtained using 5 mg of graphene sorbents showed better purification, and that is why this was the selected amount in the optimization study (Figure 1).

In addition, rGO showed slightly better results than S-GF, which can be explained by its higher BET surface area and a lower stacking of graphitic layers (*N*_L_ = 12 vs 32 for S-GF). These properties revealed the main role in improving the purification capacity. Therefore, cleanup set 5 (CL5) with 5 mg rGO, 150 mg MgSO4, 50 mg C18, and 50 mg PSA was chosen for further studies.

### 3.3 Method Validation

The *Capsicum* cultivar extract spiked with 12 BFRs at six concentration levels (0.10, 0.38, 0.75, 1.13, 1.5, and 1.88 μg/kg) was prepared for the establishment of the matrix-matched calibration curve. The analytical parameters such as linearity, LOD and LOQ, recoveries, matrix effects, precision, and uncertainty were studied (Table 2). Good linearity was observed, with the coefficient of determination >0.99. The LOD and LOQ obtained by regression analysis were between 0.10–0.25 and 0.35–0.82, respectively. The recovery of the 12 BFRs was studied by carrying out three extractions (*n* = 3) at three spiked levels (0.38, 0.75, and 1.13 μg/kg) chosen considering the LOQ values (1× LOQ, 1.5× LOQ, and 2× LOQ), thus covering the range of LOQ values. For each level, the extraction of BFRs in the *Capsicum* sample and the determinations were performed in triplicate under optimal experimental conditions. The mean recoveries between 90 and 108% were achieved. The precision was evaluated through intra- and inter-day studies, and the results were less than 14% RSD. The uncertainty was also evaluated for the 12 BFRs and was in the range between 4.9 and 18.4%, following the requirement (50%) of the EU guidance document SANTE/11813/2017.3. The details of the uncertainty studies are summarized in Table S2 in Supporting Information. In terms of the matrix effect, most of the compounds show a positive effect, resulting in a signal enhancement. Signal suppression was only observed for three compounds (PBEB, BDE47, and BDE183), with values ≤ −20%, which showed a very low suppression effect. The enhancement effect was observed for the remaining compounds and between values (19–35%). The use of these graphene-type materials in the cleaning step allowed for moderate matrix effect values. The opposite was demonstrated by Fernandes et al.,^1^ who reported a much higher matrix effect using a classical QuEChERS extraction method using commercial sorbents. The method demonstrated good accuracy, precision, and robustness and can entirely comply with the detection requirements for BFR.

**Table 2:** Method Validation Parameters

	retention time	*R*^2^	LOD μg/kg	LOQ μg/kg	mean recovery %	matrix effects %	precision %		uncertainty %
analyte	min						intra-day	inter-day	
TBECH	13.406	0.9960	0.15	0.50	95	24	13	9	6.8
BDE28	14.808	0.9981	0.10	0.35	108	35	9	9	12.5
PBT	15.017	0.9932	0.20	0.65	105	32	3	8	18.1
PBEB	15.207	0.9973	0.15	0.50	93	–10	4	9	12.2
BDE100	16.325	0.9984	0.11	0.38	104	32	9	9	13.3
BDE47	16.626	0.9927	0.25	0.82	99	–20	7	8	15.4
TBB	18.466	0.9981	0.12	0.42	94	28	7	8	4.9
BDE99	19.212	0.9945	0.13	0.43	96	29	8	11	15.9
BDE154	21.405	0.9984	0.12	0.39	99	32	8	10	18.4
BDE153	23.265	0.9956	0.19	0.64	99	22	14	9	9.6
BDE183	30.095	0.9960	0.18	0.61	90	–18	10	9	13.6
BTBPE	34.059	0.9955	0.14	0.45	92	19	10	8	14.4

### 3.4 Application to *Capsicum* Cultivar Samples

The optimized and validated QuEChERS-graphene dispersive sorbent-GC method was applied to the analysis of the BFR in 20 *Capsicum* samples that were purchased at the local market and which were packaged in 100 g plastic containers. The samples were extracted and analyzed in triplicate. The attained results demonstrate the absence of PBDE in all the samples. However, PBT was quantified in two samples at a concentration of 0.71 and 0.79 μg/kg. The results showed RSD between 5 and 11%. Confirmation of the presence of PBT in the two samples was achieved by GC–MS and GC/MS/MS.

### 3.5 Comparison with Other Published Analytical Methods

To the best of our knowledge, this is the first method reported for the simultaneous analysis of 12 BFRs in *Capsicum annum* L. matrices, and that is why comparison with other works is more difficult. Table S3 presented in the Supporting Information summarizes some studies on BFR analysis in food. A comparison of the present study with the only analytical method based on QuEChERS methodology for the extraction and determination of BFRs in *Capsicum* cultivars was performed,^1^ and the present method showed significantly better recoveries for all the BFRs (90–105%) in comparison with 66–104% for the published work.^1^ Furthermore, the LOD (0.10–0.23 μg/kg) of the developed method was significantly lower than that of the above mentioned method (1.4 and 9.3 μg/kg).^1^ We can also add a comparison of this method with others performed in BFRs and red fruit samples,^2^ and we also concluded that the analytical parameters (recoveries, LOD, and LOQ) were improved in this method. Other works on different food matrices using other extraction techniques also showed lower extraction efficiency for BFRs than the present method and higher RSD.^43^ Other studies, mostly in fish samples, are also reported in the literature.^44−47^ Comparisons are complicated because fish and related samples present different compositions when compared with our sample (*Capsicum* cultivars), namely in the amount of fat. However, comparing the analytical parameters, this new approach presented better values of recoveries, LOD, and LOQ.

In this study, we presented a novel way to cleanup the *Capsicum* cultivars using a QuEChERS approach coupled with a cleanup step adding lower amounts of graphene-type material to the dispersive sorbents. Due to the combination of the classic sorbent’s properties with graphene-type materials, they were capable of removing complicated interferences present in the *Capsicum* cultivars with higher effectiveness. The major potential of this methodology is that, when compared with the classical extraction method, the studied graphene materials added an extra cleaning to the extract, showing excellent purification performance with lower amounts of sorbents not influencing the high extraction efficiency. We also conclude that a complete characterization of the materials used as sorbents evidenced in this work is crucial and will allow advances in the development of methods. rGO provided the best results and presented the lowest number of layers and a high BET surface area, which led to the conclusion that these properties are the ones that allowed better performances.

The results revealed that this is a simple, sensitive, quick, robust, and effective modified QuEChERS method for the detection of BFR residues in *Capsicum* cultivar samples. As a promising cleanup material, graphene-type materials have future application potential to overcome hurdles associated with challenging matrices in detecting multiple contaminants in different samples.

## Supporting Information Available

Material characterization; analytical study; and comparing methods with the literature (PDF)
