# Supplementary material for: Graphene-Type Materials for the Dispersive Solid-Phase Extraction Step in the QuEChERS Method for the Extraction of Brominated Flame Retardants from Capsicum Cultivars
Source: J Agric Food Chem. 2023 Feb 16;71(8):3898–905. doi: 10.1021/acs.jafc.2c07873 (PMC9983006; doi:10.1021/acs.jafc.2c07873)
Supplement: Supplementary file 1 — jf2c07873_si_001.pdf [file jf2c07873_si_001.pdf]

Supporting information

Graphene-type materials for the dispersive solid-phase extraction step in QuEChERS method for extraction of brominated flame retardants from *Capsicum* cultivars

Virgínia Cruz Fernandes[a✉],Valentina F. Domingues[a], Marta S. Nunes[b], Renata Matos[b], Iwona Kuźniarska-Biernacka[b], Diana M. Fernandes[b], Antonio Guerrero-Ruiz[c], Inmaculada Rodríguez Ramos[d], Cristina Freire[b], Cristina Delerue-Matos[a]

[a] REQUIMTE/LAQV, Instituto Superior de Engenharia do Porto, Instituto Politécnico do Porto, Rua Dr[o] António Bernardino de Almeida, 431, 4249-015 Porto Portugal

[b] REQUIMTE/LAQV, Departamento de Química e Bioquímica, Faculdade de Ciências, Universidade do Porto, Rua do Campo Alegre s/n, 4169-007 Porto, Portugal

[c] Dpto. Química Inorgánica y Técnica, Facultad de Ciencias UNED, Senda del Rey 9, 28040 Madrid, Spain.

[d] Instituto de Catálisis y Petroleoquímica, CSIC, Cantoblanco, Marie Curie 2, 28049 Madrid, Spain.

[✉]Author to whom correspondence should be addressed:
Virgínia Cruz Fernandes

REQUIMTE/LAQV, Instituto Superior de Engenharia, Instituto Politécnico do Porto, Rua Dr[o] António Bernardino de Almeida, 431, 4200-072 Porto Portugal

Tel. + 351228340537

Email. virginiacruz@graq.isep.ipp.pt/vircru@gmail.com

Figures and Tables

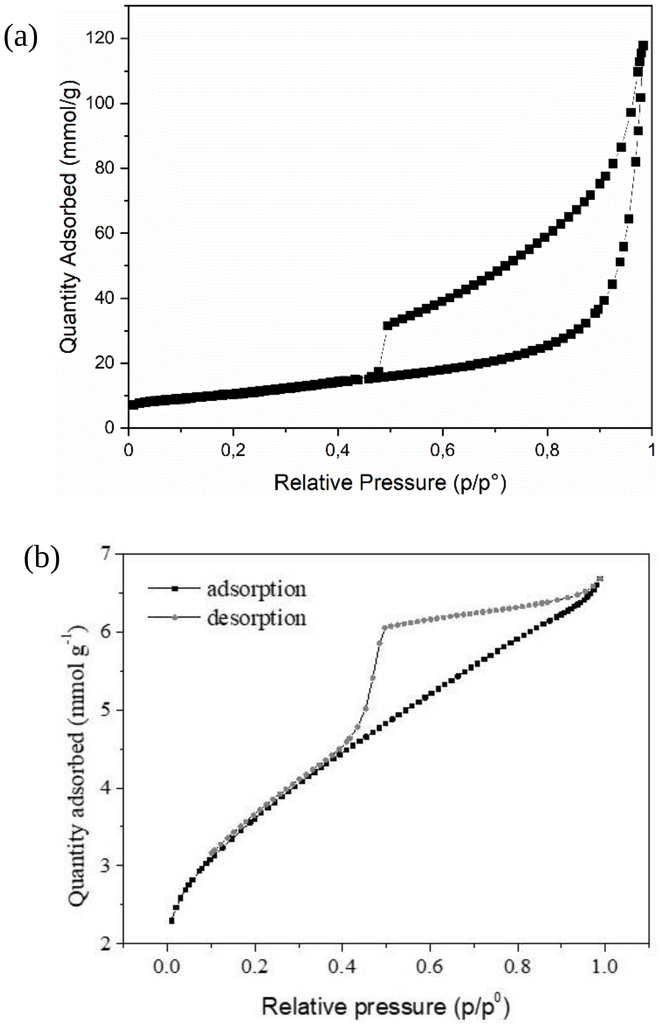

Figure S1. N_2 adsorption-desorption isotherm for rGO (a) and S-GF (b).

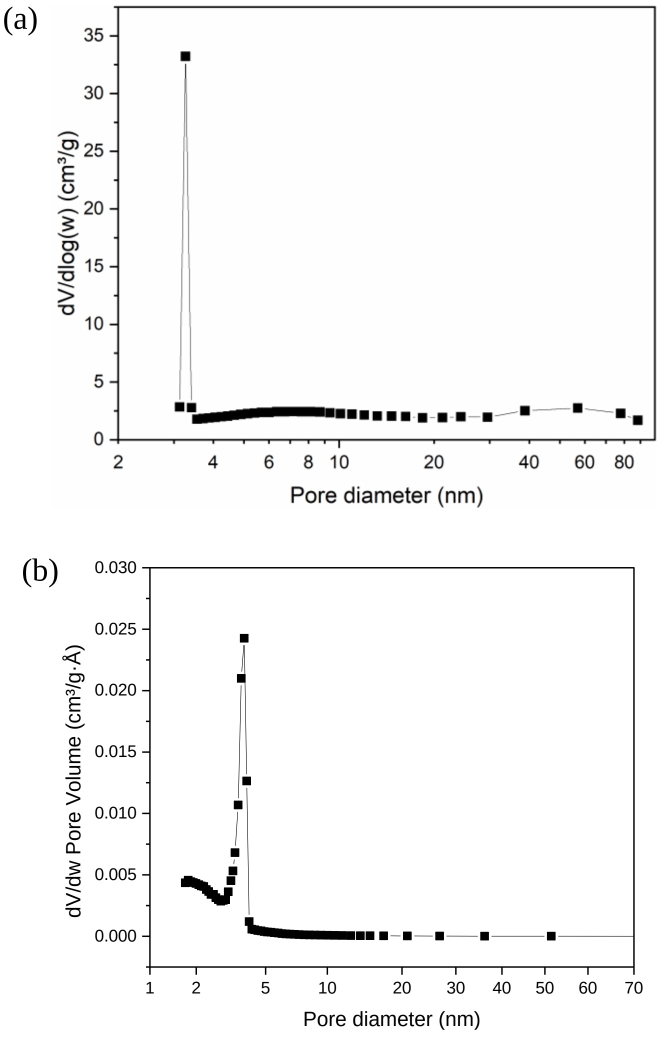

Figure S2. BJH pore size distribution curve for rGO (a) and S-GF (b).

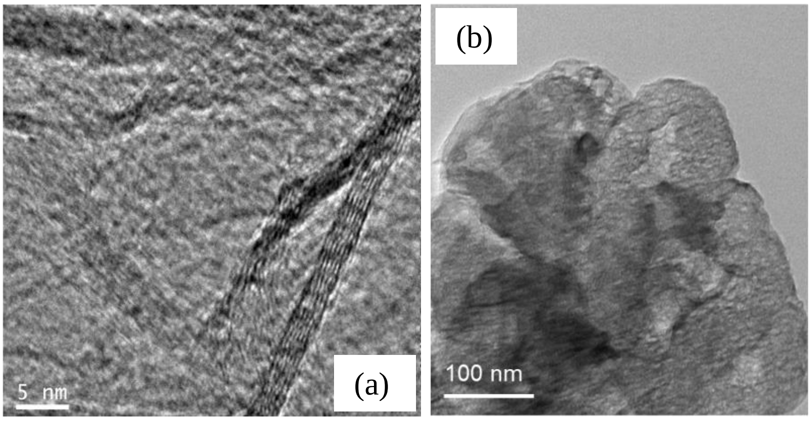

Figure S3. TEM micrographs for rGO (a) and S-GF (b) ((Matos et al., 2021; Ramirez-Barria et al., 2019)).

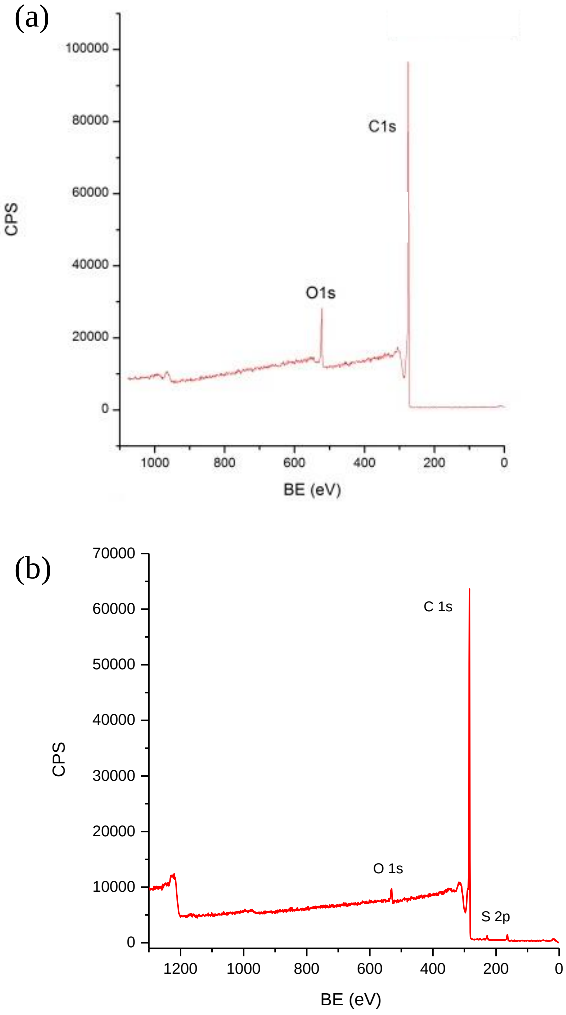

Figure S4. XPS survey for rGO (a) and S-GF (b).

Table S1. Interlayer distance ($d_{(002)}$), estimated number of layers (N_L), surface area (S_{BET}), Raman I_D/I_G ratio and C, O and S content (from XPS) of graphene-type materials (adapted from (Matos, et al., 2021; Ramirez-Barria, Fernandes, Freire, Villaro-Abalos, Guerrero-Ruiz, & Rodríguez-Ramos, 2019).

**Material**	**$d_{(002)}$ / nm**	**N_L**	**S_{BET} / m^2 g^{-1}**	**I_D/I_G**	**C / %**	**O / %**	**S / %**
**rGO**	0.34	12	867	0.63	93.0	7.0	-
**S-GF**	0.34	32	284	1.72	97.5	1.7	0.8

Table S2. Summary of uncertainties calculated.

Analyte	u_{cm}	$u_{r,repro}$ (%)	B_r (%)	$u_{r,cm}$ (%)	$u_{r,ref}$ (%)	$u_{r,tot}$ (%)	$U_{r,tot}$ (%)
TBECH	0.061	13%	-3.367	0.307	0.289	3.395	6.8
BDE28	0.063	9%	-6.217	0.317	0.289	6.232	12.5
PBT	0.191	3%	-8.983	0.956	0.289	9.039	18.1
PBEB	0.119	4%	-6.083	0.594	0.289	6.119	12.2
BDE100	0.150	9%	-6.600	0.748	0.577	6.668	13.3
BDE 47	0.136	7%	-7.683	0.680	0.289	7.719	15.4
TBB	0.104	7%	-2.400	0.518	0.289	2.473	4.9
BDE99	0.061	8%	-7.933	0.303	0.289	7.945	15.9
BDE154	0.091	8%	-9.167	0.457	0.577	9.196	18.4
BDE153	0.013	14%	-4.767	0.064	0.289	4.778	9.6
BDE183	0.036	10%	-6.767	0.178	0.289	6.776	13.6
BTBPE	0.131	10%	-7.167	0.657	0.289	7.203	14.4

Table S3. The comparison of the developed method with some reported methods.

Extraction method	Materials use in the extraction	Sample	Analysis	Sample	Limits	RSD %	Recoveries %	Ref
QuEChERS + dSPE	Graphene materials: rGO S-GF	Capsicum cultivars	GC-ECD GC-MS	Capsicum cultivars	LOD: 0.10 – 0.23 µg/kg	14	90-105	This research
QuEChERS + dSPE	Comercial Sorbents	Capsicum cultivars	GC-ECD GC-MS	Capsicum cultivars	LOD: 1.4-9.3 µg/kg	20	66-104	1
QuEChERS + dSPE	Magnetic nanoparticles	Red fruits	GC-MS	Red fruits	LOD: 0.7-9.9 µg/kg	20	65–141	2
ultrasonication and vacuum assisted extraction / QuEchERS	Comercial QuEChERS	Protein-food samples	GC–ECNI-MS	Protein-food samples	LOQ: 0.008- 0.04 ng/g dw	24%	66–135	43
QuEchERS / Silica mini column cleanup	Comercial QuEChERS	fish	GC–MS/MS	fish	LOQ: 0.005–1 µg kg−1	16%	70–119	44
QuEChERS/ acidic Extrelut-NT3 column connected on top of a silica	Comercial QuEChERS and Columns	Fish and Shellfish Tissues	GC-EI-MS/MS	Fish and Shellfish Tissues	-	20%	66–118	45

Cartridge/gel permeation chromatography
